# 磁性共价有机骨架的制备及应用——仪器分析开放性实验教学实践

**DOI:** 10.3724/SP.J.1123.2024.05018

**Published:** 2025-06-08

**Authors:** Dandan JIANG, Qiong JIA

**Affiliations:** 1.内蒙古民族大学化学与材料学院，内蒙古 通辽 028000; 1. College of Chemistry and Materials Science，Inner Mongolia Minzu University，Tongliao 028000，China; 2.吉林大学化学学院，吉林 长春 130012; 2. College of Chemistry，Jilin University，Changchun 130012，China

**Keywords:** 开放性实验, 仪器分析, 磁性共价有机骨架, 羟基多氯联苯, 液相色谱, open experiment, instrumental analysis, magnetic covalent organic framework, hydroxylated polychlorinated biphenyl, liquid chromatography

## Abstract

针对当前有机合成实验在性质研究方面的不足以及仪器分析实验方法相对独立、缺乏整合性的现状，我们设计了一个本科生开放性实验。本实验将有机合成与仪器分析实验进行结合，构建了一个包含材料合成与表征、羟基多氯联苯的吸附性能及检测方法探究的本科生创新实验。首先，采用正硅酸四乙酯对Fe_3_O_4_进行处理，引入3-氨基丙基三甲氧基硅烷制备氨基改性的Fe_3_O_4_。随后，加入三甲酰氯和对苯二胺单体用于制备磁性共价有机骨架（MCOF）。本实验利用红外光谱仪和热重分析仪对MCOF的表面基团和热稳定性进行了表征。最后，将MCOF应用于辽河水样中羟基多氯联苯的吸附，并结合液相色谱法进行检测。在实验过程中，学生不仅能够掌握羟基多氯联苯的分离和检测方法，而且能够对羟基多氯联苯在辽河水样品中的含量情况有所了解。通过实验结果的分析和讨论，使学生深刻地认识到仪器分析实验在环境监测中的重要地位。这一系统的实验流程，不仅能够提高学生综合运用无机化学、有机化学和仪器分析基础知识的能力，而且有助于提高学生分析问题、解决问题的能力及实践操作能力。同时，这一开放性实验的开展有助于提高实验室仪器设备的利用率，充分发挥实验室现有资源的作用。

## Abstract (en)

Organic synthesis experiments are often deficient and involve relatively independent experimental instrumental-analysis methods that lack integration. Accordingly， an open-instrument-analysis experiment was designed to address these shortcomings； this experiment combines organic synthesis with instrumental analysis to deliver an innovative undergraduate experiment that includes material preparation and characterization， investigating the adsorption performance of hydroxylated polychlorinated biphenyl， and exploring detection methods. First， Fe_3_O_4_ was treated with tetraethyl orthosilicate， after which 3-aminopropyltrimethoxysilane was introduced to prepare amino-modified Fe_3_O_4_. Trimesoyl chloride and *p*-phenylenediamine are then added as monomers to synthesize a magnetic covalent organic framework （MCOF）. The surface groups and thermal stability of the MCOF were then characterized using Fourier-transform infrared spectroscopy and thermogravimetric analysis， after which the MCOF was used to determine hydroxylated polychlorinated biphenyl in Liaohe River water samples using liquid chromatography. During this experiment， students master separation and detection methods for hydroxylated polychlorinated biphenyl， while also learning about its levels in the Liaohe River. Students will recognize the important role that instrumental analysis plays in environmental monitoring by analyzing and discussing the experimental results； they will also improve their abilities to comprehensively apply basic inorganic chemistry， organic chemistry， and instrumental-analysis knowledge， while also improving their abilities to operate， analyze， and solve problems. Implementing this open experiment will help to improve the use of laboratory equipment and fully utilize existing laboratory resources.

仪器分析是化学专业以及许多相关学科（如生物科学、材料科学、环境科学等）的公共基础核心课程之一。这门课程的主要目的是让学生掌握现代仪器分析的基本原理、方法和技术，以便能够在实际的科学研究和工业生产中应用这些技术^［1，2］^。仪器分析实验能够帮助学生掌握各种现代仪器的使用技能，如色谱仪、光谱仪、质谱仪等。将新型纳米材料的合成及表征与仪器分析实验结合，通过开放性实验的形式来培养学生的创新意识和研究能力，是一个富有前瞻性和实践性的教学策略。通过参与开放性实验，学生能够全面提升自己的综合素质和创新能力，并学会与他人协作和分享，为未来的学术和职业发展奠定坚实的基础^［3，4］^。这种教学模式能够充分展示仪器分析在化学研究中的核心作用，同时克服大型仪器成本高、难以广泛使用的局限。

羟基多氯联苯（OH-PCBs）是一类具有高毒性的持久性有机污染物，其生物累积性导致了全球环境污染^［5，6］^。OH-PCBs能通过食物链累积在生物体内，对生态系统和人类健康构成潜在威胁。水生环境是众多持久性污染物的主要蓄积库之一，因此对水体中的OH-PCBs进行定期监测和评估具有重要意义。目前，测定OH-PCBs最常用的方法之一是液相色谱法^［7］^。然而，OH-PCBs在环境中的含量通常较低。液相色谱法虽然具有高灵敏度和高分辨率等优点，但其检出限仍然是一个需要考虑的因素。因此，对样品进行分离预富集是提高OH-PCBs检测效率和准确性的重要步骤。

共价有机骨架（COF）是一类由轻元素C、H、B、N、O等组成的晶体多孔材料^［8，9］^。它具有密度低、结构可调、功能化和稳定性好的优势，被认为是一种有前途的吸附材料^［10，11］^。磁性共价有机骨架（MCOF）是一种将COF与磁性基质组合起来的新型纳米复合材料。MCOF可以通过其孔道对OH-PCBs进行选择性吸附，并通过调节孔道的大小和形状来实现对不同目标物质的分离。同时，MCOF的磁性使得它可以方便地通过磁场进行分离和回收，提高了吸附剂的重复使用性和效率^［12，13］^。

笔者所在团队结合科研工作设计了一个开放性实验，将MCOF的制备表征及其在OH-PCBs分离富集中的应用引入仪器分析实验教学。由教师引导学生查阅相关文献，设计实验方案并细化实验步骤。实验过程包含材料制备、表征、吸附性能探索及OH-PCBs的液相色谱检测，涉及多种合成及仪器使用方法。本实验可以让学生接触到最新的科研动态和技术进展，有助于学生了解当前化学领域的研究热点和趋势，为未来的学习和研究打下坚实的基础。通过实验将绿色化学、环境友好型化学等可持续发展理念植根于学生的内心，对于强化学生的环保意识和责任感具有重要意义。

## 1 实验部分

### 1.1 试剂、材料与仪器

羟基多氯联苯标准品（2-OH-CB 124）购自AccuStandard公司（美国）。四氧化三铁（Fe_3_O_4_）、乙酸乙酯（EA）、3-氨基丙基三甲氧基硅烷（APTES）、三甲酰氯（TMC）、对苯二胺（PPD）、氨水（NH_3_·H_2_O）、氢氧化钠（NaOH）、乙腈（ACN）、正硅酸四乙酯（TEOS）、乙醇和正己烷购自上海阿拉丁试剂公司。

KQ-200KDB型超声波清洗仪（青岛聚创环保集团有限公司），JJ-1型磁力电动搅拌器（郑州南北仪器有限公司），NICOLET 5700傅里叶变换红外光谱仪（FT-IR，赛默飞世尔，美国），Q500型同步热重分析仪（TG，珀金埃尔默，美国）。

### 1.2 仪器检测条件

红外光谱：采用溴化钾压片法制备固体样品，仪器分辨率为4.0 cm^‒1^，扫描次数32，波数范围为4 000～500 cm^‒1^。热重分析：仪器升温速率为10 ℃/min，温度范围为25～800 ℃，吹扫气氛为N_2_。

色谱分析采用安捷伦1200型液相色谱仪（配置紫外可见检测器）。采用SHISEIDO CR 1∶4柱（150 mm×2.0 mm， 3.5 mm），将流动相的流速设置为0.25 mL/min。将10 μL样品自动注入液相色谱柱，温度保持在30 ℃。流动相为ACN（A）和水（B）。梯度洗脱程序如下：0～2 min，85%A；2～8 min，85%A～10%A；8～12 min，10%A；12～12.1 min，10%A～85%A；12.1～20 min，85%A。

### 1.3 标准样品及辽河水样品处理

标准样品的配制：2-OH-CB 124储备液（1 000 ng/mL）由正己烷作为溶剂制得。

辽河水样品处理：在辽河流域的不同地点采集水样，确保采集的水样具有代表性。采用NaOH溶液将样品pH调节至11，将碱化后的水样经0.45 μm微孔滤膜过滤，过滤后样品于4 ℃冰箱中备用。

### 1.4 磁性吸附剂的制备

MCOF的制备流程如图1所示。将Fe_3_O_4_ （1.0 g）分散在含有乙醇（40 mL）、H_2_O（10 mL）、NH_3_·H_2_O（0.8 mL）和TEOS（0.5 mL）的混合溶液中，在40 ℃下搅拌1 h。随后加入APTES（1 mL），在25 ℃下搅拌1 h，获得Fe_3_O_4_@NH_2_。

**图 1:**
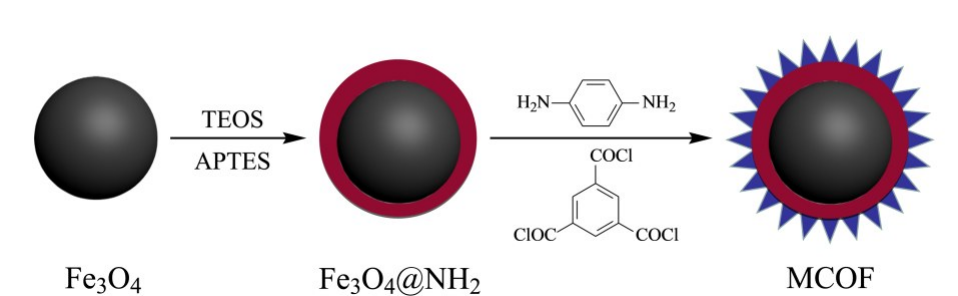
MCOF的制备流程图

将Fe_3_O_4_@NH_2_ （1 g）分散于含有TMC（1.325 g）的EA（30 mL）溶液中，在0 ℃下搅拌1 h。随后加入含有TMC（1.06 g）和PPD（0.32 g）的EA（30 mL）溶液，在25 ℃下搅拌3 h，得到MCOF。

### 1.5 吸附过程

将MCOF（30 mg）分散在样品溶液（1 mL）中，超声处理30 min后，采用磁铁分离MCOF。将上清液倾倒，用正己烷/EA溶液（1∶1，v/v）作为洗脱液，超声处理10 min。经过磁性分离后，得到的上清液进行液相色谱测定。

## 2 结果与讨论

### 2.1 MCOF的表征

采用FT-IR对MCOF的化学结构进行分析。如图2a所示，位于580 cm^‒1^处的典型吸收峰属于Fe‒O的伸缩振动。位于2 926 cm^‒1^和2 854 cm^‒1^处的吸附峰属于芳香环的C‒H振动，而3 438 cm^‒1^处的吸收峰归属为O‒H/N‒H振动。另外，羧基和酰胺的C=O特征吸收峰出现在1 713 cm^‒1^和1 651 cm^‒1^处。FT-IR结果证明了MCOF的成功制备。

**图 2:**
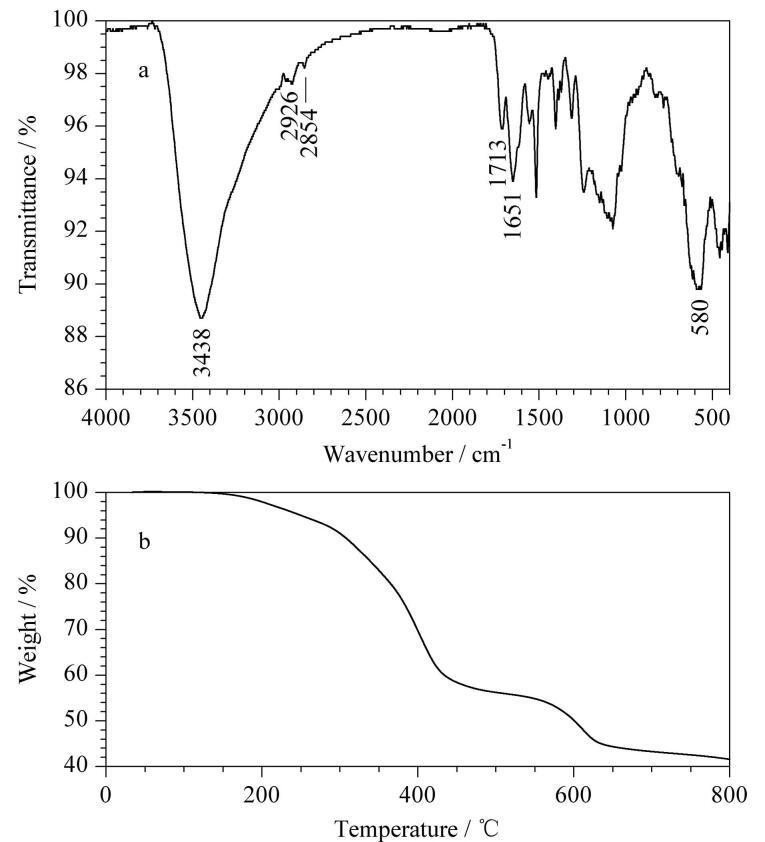
MCOF的（a）红外表征图和（b）热重分析图

采用TG对MCOF的热稳定性进行分析。如图2b所示，在200 ℃之前，MCOF的重量基本没有损失，符合吸附实验所需。在200～430 ℃的温度范围内，MCOF质量的减少主要是由于COF包覆层的分解引起的。在430～630 ℃的温度范围内，MCOF的质量损失是由于氨基的分解引起的。在630～800 ℃内，Fe_3_O_4_的晶格被破坏并逐渐转化为*γ*-Fe_2_O_3_。

### 2.2 吸附条件优化

本实验考察了样品pH值、MCOF用量和吸附时间对2-OH-CB 124吸附效率的影响。首先验证了在pH值3～11范围内对2-OH-CB 124回收率的影响（图3a）。结果表明MCOF对2-OH-CB 124存在吸附能力，且在pH值为11时回收率最大。因此，确定最佳的pH值为11。

**图 3:**
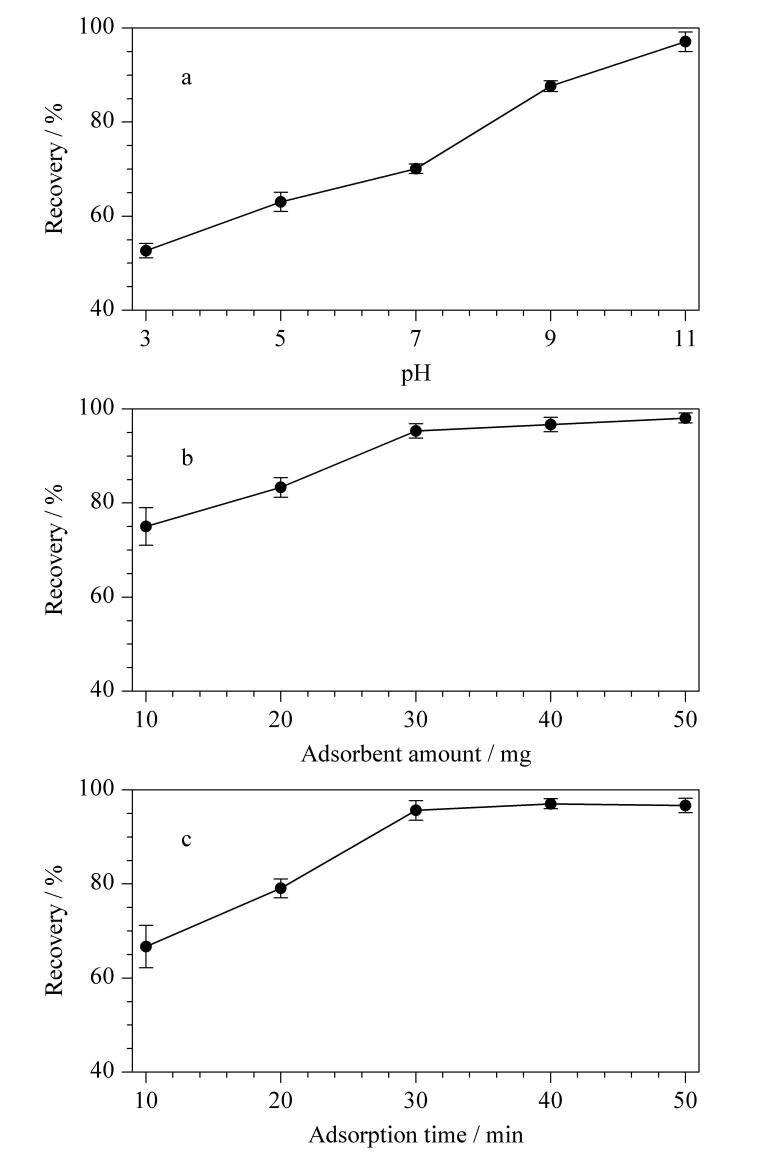
吸附条件的优化（*n*=3）

考察了不同的MCOF用量对2-OH-CB 124回收率的影响。2-OH-CB 124的回收率在MCOF用量为30 mg时基本保持不变（图3b）。考察了不同吸附时间（10～50 min）对2-OH-CB 124回收率的影响。从图3c中可以观察到，MCOF在30 min内可以迅速达到吸附平衡。因此，本实验最终确定的MCOF用量为30 mg，吸附时间为30 min。

### 2.3 羟基多氯联苯的标准曲线绘制

在最佳实验条件下，测定不同质量浓度的2-OH-CB 124标准溶液（1、5、10、30、50和70 ng/mL）以获得本方法的标准曲线。2-OH-CB 124的线性范围为1～60 ng/mL，相关系数为0.997 3，检出限（LOD， *S/N*=3）为0.34 ng/mL，定量限（LOQ， *S/N*=10）为1.12 ng/mL。以上结果表明本方法能够检测痕量2-OH-CB 124。

### 2.4 辽河水样品的测定

本实验选取辽河水作为实际样品，对不同采样点的3种水样品进行分析，结果表明，样品中均检测到2-OH-CB 124，含量分别为2.57、3.87和7.58 ng/mL。让学生通过加标回收试验测定水样品中2-OH-CB 124的回收率。2-OH-CB 124的加标水平分别为1.0、5.0和10.0 ng/mL。比较加标前后的色谱图（图4），计算加标回收率。回收率实验结果见表1所示。

**图 4:**
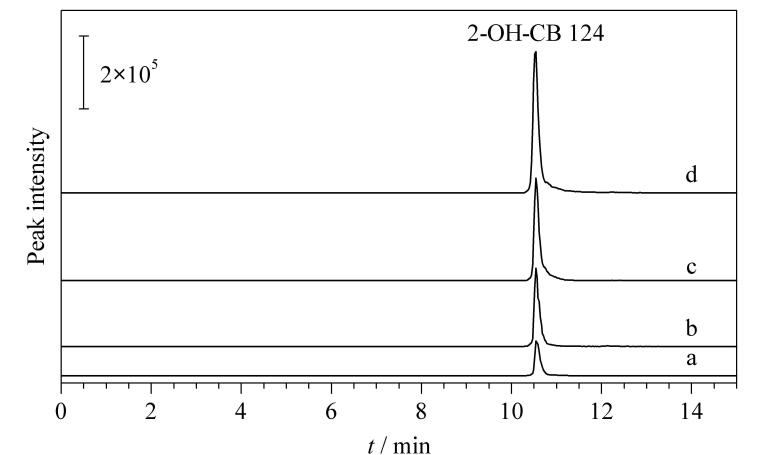
不同加标水平下实际样品的色谱图

**表 1:** 实际样品中2-OH-CB 124在3个水平下的加标回收率（*n*=3）

Water sample	Measured value/（ng/mL）	Spiked/ （ng/mL）	Recovery/%
1	2.57	1.0	90.5±0.2
		5.0	93.5±0.1
		10.0	89.8±0.5
2	3.87	1.0	99.5±0.1
		5.0	100.5±0.4
		10.0	88.5±0.6
3	7.58	1.0	92.7±0.7
		5.0	97.5±0.3
		10.0	96.8±0.4

## 3 实验开展

### 3.1 实验的组织实施

本实验安排为16学时，可分成2次课（各8学时）进行。第1次课：磁性吸附剂的合成；第2次课：红外光谱分析、热重分析、羟基多氯联苯的吸附条件优化、液相色谱测试、实验结果讨论与总结。

### 3.2 实验问题反馈及注意事项

①本实验的材料制备部分所需时间较长，每位学生需单独完成此部分，在空闲时间教师可以引导学生查阅与实验相关的文献资料，了解实验背景知识和最新研究进展。②表征测试部分涉及红外光谱仪和热重分析仪，建议2人一组分工协作完成实验。在仪器使用前，学生应自行阅读并理解仪器的使用说明书，教师授课时应充分讲解基本原理和工作机制。③羟基多氯联苯的吸附条件优化及液相色谱测试涉及步骤较多，应该根据实际操作情况来进行合理的学生分配。

实验结束后，收集学生评价和收获反馈，结果充分展示了综合开放性实验对学生学习兴趣的激发和综合能力提升的重要性。学生表示通过对前沿文献的调研，了解到学科领域的最新研究成果和发展趋势。这不仅拓宽了他们的视野，还激发了他们对未来科学研究的向往和追求。通过运用各种分析表征技能，如光谱分析、色谱分析等，不仅锻炼了他们的实践能力，还提高了他们的综合分析能力。因此，我们应该继续加强综合创新实验的教学和实践，为学生提供更多更好的学习机会和平台。

## 4 结论

本实验设计了MCOF的制备表征及其应用于辽河水样品中羟基多氯联苯的吸附与检测。实验涵盖了从磁性吸附剂的合成到羟基多氯联苯液相色谱检测的全过程，涉及无机化学、仪器分析和有机化学等多个学科领域。实验准备阶段，学生需要查阅大量相关文献，了解磁性吸附剂的研究进展和实验方法。通过这一阶段的学习，学生的科研论文查阅能力将得到锻炼和提升。实验过程中，学生还需掌握多种实验技能，如材料合成、表征方法（如FT-IR、TG等）、吸附性能测试（如羟基多氯联苯的吸附实验）等。同时，实验要求学生具备谱图解析和实验数据绘图的能力。最后，通过对实验数据的处理和分析，使学生能够准确地了解材料的性能和结构，进而提高科研论文的撰写水平。

本实验操作简单，能够使学生在较短时间内掌握实验技巧，大大降低了因操作复杂而导致实验失败的风险。实验提供了多种结构性质表征方法，这些方法不仅丰富了学生实验技能的学习，还有助于学生从多个角度理解和分析实验结果，提高他们的综合分析能力。本实验包括有机合成与仪器分析两个独立且互补的实验模块。有机合成实验侧重于化学合成的基本技能，如反应条件控制、产物分离纯化等；仪器分析实验侧重于现代分析技术的应用，如光谱仪等仪器的操作。两个独立模块的组合可以形成一个完整的科学研究过程，对促进学生全面了解科研工作的流程和要求具有重要的意义，也为他们未来从事科研工作打下了坚实的基础。
